# Phthisis Pulmonalis

**Published:** 1837

**Authors:** 


### REVIEWS AND BIBLIOGRAPHICAL NOTICES.

### Art. VIII.—Phthisis Pulmonalis.

*Pathological Researches on Phthisis.* By C. A. Louis, M. D., Physician to the La Pitie Hospital of Paris, etc., etc. Translated from the French, with notes and additions, by Charles Cowan, M. D., of England; revised and altered by Henry J. Bowditch, M. D., of Massachusetts. Boston, Hilliard, Gray & Co., 1836. pp. lxxii.—550.

M. Louis is undoubtedly one of the most acute and indefatigable observers of the age. Scarcely fifty years old, standing as he does at the very pinnacle of his profession, he may be emphatically called the prince of physicians. His works, so far as they have reached us, are all distinguished for their originality and depth on research, and will forever remain as standards on the subjects of which they treat; they form, indeed, an era in the history of medicine; and whilst they confer upon their author an enviable immortality, they may boldly challenge, as far as accuracy of observation is concerned, a comparison with any scientific productions, either of ancient or modern times.

How is it that M. Louis has attained his present elevated position in the ranks of his profession? Was it by an attentive and laborious study of the works of his predecessors and cotemporaries? Is it by extraordinary mental endowments? Is it by genius? Yes! but not that genius spoken of by the lexicographer and the man of letters; not that sprightliness of intellect, that quickness of perception, that intuitive power of comprehension, usually implied by this term; but the genius of industry, of patient perseverance, of calm and rigorous observation. Such is the genius of M. Louis; such the genius which has guided him in his researches; such the genius by which he has obtained an imperishable name.

Commencing the study of medicine at an early period of his life, he was soon struck with the contradictory theories of his predecessors, and became fully convinced, that, to arrive at useful results, it was necessary to lay aside his books, and to interrogate nature, by diligent and careful observation of her phenomena, at the bed-side of the sick; followed up by a patient examination of the body after death. Until the age of thirty-three, he practised his profession in Russia; but about the end of this period, namely in 1821, accidental circumstances brought him to Paris, the great emporium of medical science. Here, attracted by the eloquence of the "gastro-enteritic" magician, the celebrated Broussais, he eagerly studied his writings, and assiduously attended his prelections, his practice, and his post-obit investigations. But his hopes were again doomed to be disappointed. In his new master, he beheld at first the very beau-ideal of a medical philosopher. But day after day, the painful fact obtruded itself upon his active and inquiring mind, that Broussais was not quite so perfect as he had been originally led to suppose. He soon found that the great reformer—he who bid fair at one time to light up the whole medical horizon—had himself his weak points; that, although he was fully competent to oppose the errors of others, he often failed in demonstrating himself to be right, save in his own conceit.

Tired thus at getting knowledge second-handed, and disgusted at seeing nature so often misinterpreted, Louis resolved to devote himself exclusively to observation, and with this view he forthwith entered the charity hospital of Paris, as a *clinical clerk.* In this capacity he continued nearly seven years, consecrating his best talents and efforts to the improvement of his profession. Investigating every case of interest that came under his notice, he conducted his researches with so much patience and minuteness as to bring upon him often the sneers and ridicule of others, less diligently devoted than himself to the pursuit of knowledge. All his time was spent at the hospital, in examining the patients, writing down their symptoms, and inspecting such as died under treatment. His work on Phthisis, published at Paris in 1825, was the result of the first three years of his connexion with this institution, being drawn up entirely from materials derived from its inmates. The number of patients admitted into the wards of the hospital during the above-mentioned period was nearly two thousand, of whom three hundred and fifty-eight died. Out of these last, one hundred and twenty-seven were cases of pulmonary consumption. These cases, which were carefully studied, both during life and after death, form the basis of the present work. To give the reader some idea of the care with which the pathological facts were drawn up, it is only necessary to state that M. Louis never devoted less than two hours to his necroscopic examinations; and this practice, we are informed, he still continues at the present moment. For the last five years he has been physician to La Pitie, one of the principal hospitals of Paris; and here, heaven grant, he may long remain, directing his pupils in the path of observation and induction, enlightening the profession by his writings, and benefiting mankind by his labors and discoveries.

With this sketch of the author, we proceed to the book, of which the reader must be anxious, by this time, to obtain some knowledge. In the review which we are about to present, it will be our object to be strictly analytical; premising, however, that, where occasion may require it, we shall not hesitate to introduce such matter as may be relevant to the subject under discussion. In this way we hope to be able to fill up a few of the lacunæ, which are to be found in different sections of the work.

The work is divided into two parts; the first is devoted to the consideration of the morbid anatomy of the lungs, and of the various other organs implicated in phthisis; the second, to the exposition of the symptomatology of the disease, its diagnosis, causes, and treatment. Each part is fraught with valuable matter, and therefore demands separate consideration.

Part 1.—In the opinion of Louis, phthisis consists, *purely and entirely*, in the development of tubercles in the lungs. In all the subjects who died of this disease at La Charite, there was not a single one, says he, who did not present this lesion. This notion is by no means peculiar to the author. P. Desault, of Bourdeaux, in an essay on phthisis, published more than a hundred years ago, clearly states that tubercles constitute the essence of consumption; and the same doctrine is warmly inculcated in the immortal work of Laennec. Bayle, on the other hand, admits several kinds of phthisis, as, for example, the tubercular, the cancerous, melanotic, and granulated; and M. Broussais informs us that he has often witnessed cases of the complaint from chronic and suppurative inflammation of the lungs, in which no trace of tubercles could be detected after death. These statements are sufficiently discordant; yet I cannot but think that the restriction indicated by Desault, and so much insisted upon by Laennec and Louis, is pathologically correct. There are, indeed, properly speaking, other varieties of consumption; but there is but one species of pulmonary phthisis, and that species is dependent *exclusively* upon the presence of tubercles in the lungs.

The anatomical characters of tubercles have been so fully escribed by his two countrymen, Bayle and Laennec, that the author contents himself wih a very concise account, in which, however, many remarks are to be found peculiar to himself. In most of the cases, the tubercles existed in both lungs, and, what is remarkable, they were almost invariably more numerous, larger, and more matured at the summit than at the base of these organs. In 123 cases, he met with only two exceptions to this rule. Bonetus, Morgagni and Stark long ago remarked that the left lung is more frequently affected with tubercles than the right; and his opinion is perfectly in unison with that of Louis, though it is opposed to that of Laennec and Lombard. It is also in accordance with the experience of Dr. Morton, of Philadelphia. In 86 cases analysed by this physician, both lungs were equally affected in seven cases, the left most in 51, and the right in 28.

Tubercles occur in all the animal tissues, excepting those which form the more solid portions of the body. Nor are they confined, as was at one time presumed, to the human subject. They have been observed in many species of animals, both carnivorous and herbivorous, in birds, reptiles, and even in insects; though, as respects the latter facts, are still wanting to illustrate the subject.

The tubercular matter has been analysed by different chemists; but the most correct information that has been furnished on this point is by M. Hecht, of Strasburgh. According to this philosopher, it consists of nearly equal proportions of albumen, gelatine and fibrin. With these results, the analysis of Thenard, previously made, is strikingly at variance. This chemist found that 100 parts of crude tubercular matter contained upwards of 98 parts of albumen, the remainder being made up of muriate of soda, phosphate and carbonate of lime, and a traçe of the oxide of iron. The most interesting fact developed by the analysis of M. Hecht, is the existence of fibrin, which intimately assimilates this matter, if it does not render it identical, with the adventitious membranes of the serous and mucous textures.

Tubercular matter is deposited under several distinct forms. Of these, the most common by far is the *miliary*, from milium, a millet-seed. The tubercles in this species of the disorder resemble small rounded grains, varying in size from a pin-head to that of a pea. They are generally of a dull yellowish color; in many instances, however, they are greyish, mottled, or milky. Their consistence also varies considerably, being sometimes as hard as fibro-cartilage, whilst at other times they are soft, and almost semi-fluid. In the former case, they often exhibit, when cut, a bright vitreous aspect, not unlike ground glass. In their primitive state, the miliary tubercles are perfectly distinct from each other, but ultimately they coallesce, so as to destroy completely the original separation, resembling in this respect the confluent pustules of small pox. Large masses are thus frequently formed, of a dense, cartilaginous consistence, and of an angular, globular, ovoidal, or stellated configuration.

Cases occur, in which these bodies are surrounded by a distinct capsule, constituting the *encysted tubercle* of Bayle. The envelope is usually of a fibrous nature, and of a white greyish color; sometimes pinkish, violet, or mottled. Varying in thickness from the fourth of a line to a line, it adheres closely by its outer surface to the parenchymatous structure of the lungs, from which it can be separated only by the knife, or by forcible laceration. The contents of the cyst are commonly of a greyish-yellow color, opake, and speckled with dark points, and so long as they remain solid they are firmly attached to its inner surface. Both structures seem to have a cotemporaneous origin, and it is altogether reasonable to conclude that they are the result of a new formation, and not merely the distended walls of the air-cells of the lungs, as they are absurdly supposed to be by Dr. Carswell. This variety of tubercle is extremely rare. Louis has seen only a solitary example of it; and thus far, although we have made numerous examinations of persons who have died of phthisis, we have never witnessed an instance of it.

A third species of tubercular matter exists in the form of infiltration. It is often found round tubercular excavations, sometimes in considerable patches, of a greyish translucent appearance, more or less dense, crisp, and firm like cartilage. In its texture it is apparently homogeneous, presenting, when divided with the knife, a smooth, polished surface, in which it is impossible to perceive the slightest trace of air-cells. Occasionally the deposition has the appearance and consistence of jelly, constituting the *gelatinoid infiltration* of Laennec; a distinction which is as unnecessary as it is unscientific, as it forms merely a variety of what we have just described.

Much has been said by writers concerning crude and semi-concrete tubercles, some supposing that they are originally deposited in a solid, others in a liquid state. Without stopping to inquire into the merits or demerits of these questions, it may be remarked, that every known fact in pathological physiology is strongly corroborative of the opinion, that all tubercular matter is primitively soft, semi-liquid or gelatinous. The chemical, physical, and anatomical characters of this matter all conspire to show its identity with coagulating lymph; and if this identity be admitted, as we think it must by every one who has investigated the subject, who will contend that it is ever deposited in the solid form? Where is there an instance in which the fibrin of the blood is eliminated in a concrete state? Do we find it thus thrown out in the inflammations of the serous membranes? Is it thus deposited between the edges of a recent wound or round the fragments of a broken bone? Experience and common sense alike answer in the negative. We hold, then, that all tubercular matter, whatever may be its form, extent, or situation, is in the first instance soft, and that it becomes concrete only by the removal of the serum which is always poured out simultaneously with it.

The period required for the development of solid tubercles is so exceedingly variable that it is difficult to lay down any definite rules upon the subject. In acute phthisis, Louis says, they may reach the size of a pea in three or four weeks; in other cases they may remain small for a considerable period, sometimes for several months, although all the symptoms of the disease may be fully formed.

Various notions have been entertained by pathologists respecting the precise nature of tubercles, some of which it must be confessed are extremely crude and unsatisfactory. Hippocrates, and Aretæus, Jeaunot de Langrois, Fernal, Morgagni, and Dr. Reid, of Edinburgh, ascribe their formation to the concretion of a thick, viscid fluid, poured out by the vessels into the delicate texture of the lungs, and probably analagous to what has since been termed the coagulating lymph. Sylvius, whose works were published towards the close of the seventeenth century, imagined that they were nothing but lymphatic ganglions, which, scattered every where through the parenchymatous structure of the lungs, were rendered morbid by the effects of inflammation. This opinion was afterwards adopted, and more fully illustrated by Wepfer and Morton, and, strange as it may seem, it has received the sanction of the great Broussais and several other distinguished writers of our own day. Laennec, it is well known, believed that they are always deposited in a crude state; and similar are the views which have been expressed by several of his distinguished countrymen. The reverse of this opinion has been recently maintained, with much zeal and pertinacity, by Dr. Baron, an English physician. In his treatise on "Tubercular Diseases," published in 1822, he attempts to prove that these little bodies are essentially hydatic, and that instead of passing from an indurated to a softened state, as is asserted by Laennec, they are originally simple vesicles, filled with thin albuminous matter, which in process of time undergoes inspissation. Amidst such discrepancies of opinion, it is extremely difficult to decide which of the doctrines here referred to is most entitled to our confidence and regard. After much reflection, however, upon the subject and from careful and repeated examinations of tubercles in different organs of the body, I am constrained to believe that these structures are originally nothing but coagulating lymph, thrown out as an effect of inflammatory irritation. Leaving this topic, however, to be more fully discussed in another publication, we shall only further remark, in relation to it, that we are not singular in the doctrine which ascribes the formation of tubercles to this disease. Broussais, as has been already intimated, is one of the most strenguous advocates of this opinion; but he greatly errs, if we mistake not, in supposing that tubercles are merely diseased lymphatic ganglions. The inflammatory origin of these bodies is also maintained by Andral and Lombard. M. Louis, on the contrary, supposes that inflammation, though it may occasionally exert an influence of this kind, produces no such results in the generality of cases. We have already seen that he regards pnumonitis and phthisis as two distinct diseases, which, although they may, and often do co-exist, are entirely independent of each other.

Are tubercles ever organized? On this subject, too, much difference of opinion prevails amongst pathological anatomists. The question is but faintly alluded to in the work before us; indeed, there is scarcely any writer who has given us any satisfactory information concerning it. That tubercles are invariably organized, that is, furnished with vessels, nerves and absorbents, no one, it is presumed, will attempt to assert; but that they are thus supplied under certain circumstances does not admit of the slightest doubt. Within the last twelve months, we have examined not less than three specimens of organized tubercles, one of them occurring in the lungs, the other two in the spleen, in children under a year old. They were of the miliary kind, and numerous vessels, loaded with florid blood, could be seen shooting into them in every possible direction, many of them penetrating a considerable distance into their substance. We have never been able to trace any nerves or absorbents into these bodies, nor do we know that this has ever been accomplished by others; but as these structures are every where necessary to organization, it is reasonable to infer their existence. Another proof, but of a more indirect character, of the organic nature of tubercles, is furnished by the fact, witnessed by numerous and highly respectable observers, that these bodies are often of a yellow tint in jaundice; which is owing, no doubt, to the admixture of the coloring matter of the bile with the blood, both being simultaneously circulated through the substance of the tubercular mass.

But, be the origin and nature of these bodies what they may, whether they are nothing but diseased lymphatic ganglions, or whether they are a deposition of lymph, produced by inflammatory action, and susceptible of organization, they sooner or later become soft and fluid, the necessary consequences, it may be presumed, of their crude condition. This process is supposed by Louis and Laennec always to begin in the centre of each mass; but Andral and Carswell very justly maintain that it may commence at any part, indifferently at the centre or at the circumference. As it progresses, the tubercular matter becomes daily more soft, moist, and unctuous, until at length it acquires the ropiness and fluidity of pus. When the degeneration implicates the whole mass, it is usual to find two different kinds of matter in it; one thick, straw-colored, and inodorous, like laudable pus; the other thin, commonly tinged with blood, and mixed with small, opake, cheesy flakes. This is more particularly the case in scrofulous subjects, in whom the fluid in question often strongly resembles whey, with minute portions of curd floating in it.

Conformably with the doctrine which it has been attempted here to enforce, that tubercles are organized structures, the process of softening may be supposed to be analagous to slow suppuration, by which the heterologous tissue is gradually broken up and destroyed. Let us be understood. After tubercles have existed for some time in the lungs, they begin to act as extraneous bodies, producing irritation in the immediately circumjacent textures. This irritation, it may be presumed, is speedily propagated to the tubercles themselves, and as their vital endowments are comparatively feeble, and consequently incapable of much resistance, they soon yield to the invasion, the rapidity of their softening being always in direct ratio to the intensity of the exciting cause. Those, on the other hand, who believe that tubercles are inorganic, maintain that their softening is brought about by the agency of the surrounding tissues, alleging that they are incapable of undergoing such a change by any powers of their own. If this explanation were correct, the process should always begin at the periphery of these bodies, which, however, as was before stated, is not the case.

The softening of tubercles, according to the observations of M. Louis, takes place at very different periods; in some cases from the twentieth to the fortieth day, counting from the commencement of the disease; generally, however, not until much later. Occasionally the process goes on simultaneously over a large extent of surface, so as to break down one or two lobes; but this occurrence is extremely rare, and is confined exclusively to acute phthisis. Much more commonly, the degeneration begins at the summit of the lungs, and extends towards the base, as is shown by the fact, that if these organs be examined in this direction, we successively find, at various heights, excavations, and tubercles in different stages of softening, the more solid being always lowest in the scale.

During the progress of phthisis, the tubercular matter, after having become perfectly soft, works its way into the neighboring bronchial tubes, leaving thus not unfrequently a considerable number of *excavations*, *caverns* or *fistulous apertures*. M. Louis has never found these caverns entirely empty before the end of the third, or the beginning of the fourth month, counting from the invasion of the disease. In recent cases of this kind, the walls of the chamber are generally soft, and lined by a thin layer of lymph, which is easily separated from the surrounding parts; but in more protracted instances, the false membrane is dense, greyish, sometimes semi-cartilaginous, and from one-fourth to one-third of a line thick. Occasionally several layers are thus deposited, the one last formed being always more delicate, more easily torn, and of a more yellow color. In one-fourth of the cases there was no membrane at all.

The old caverns were sometimes surrounded by tubercular matter, or even by melanosis; and they still further differed from those which were recent in being uneven, ragged, and in communicating generally with smaller cavities. Frequently they were crossed in different directions by small bands, consisting either of condensed cellular substance, or of blood-vessels. The intersections were uneven, of variable length, narrow, from one to two lines thick, thinner in the middle than at the extremities, and frequently studded with tubercular deposits. We have seen these cords in a number of specimens; in one, preserved in the museum of the Cincinnati College, they bear a striking resemblance to the fleshy columns of the heart. In no instance did the author meet with the remains of bronchial ramifications in the interior of these tubercular excavations; and in but one case was there a fragment of pulmonary tissue.

The size of these excavations is sometimes very great. M. Louis has frequently found them as large as an apple, and in some instances even of the volume of a goose's egg. Occasionally they do not exceed the size of a hazel nut, an almond, or a walnut. In the great majority of cases the excavations occurred in both lungs; but this was not constant, for in about a sixth of the subjects whom he inspected they existed only on one side.

In recent cases, the *contents* of the excavations were thick and yellowish, like common pus; in those, on the contrary, of long standing, the matter was thin, greenish, sanious and offensive, sometimes tinged with blood, or even of a deep red color. Instead of air or pus, M. Louis found in one instance an organized fibrous body, filling up a tubercular cavern, as large as a middle sized apple.

Tubercular excavations occasionally *cicatrize.* Upon this point, however, M. Louis has left us no positive data; but of its truth abundant and undeniable evidence is to be found in the works of Laennec, Andral, Forbes, and other writers. Several examples of cicatrization of tubercular excavations have occurred to us within the last eighteen months, one of which is now contained in our museum of pathological anatomy. The healing takes place in three ways: first, by the surface of the cavity becoming a healthy membrane, the cavity itself remaining open; secondly, by the contraction of the excavation, and the gradual agglutination of its walls through the intervention of dense cellular tissue; thirdly, by an effusion of coagulating lymph, or repeated depositions upon the inner surface of the fibro-cartilaginous walls of the cavity, forming a white bluish mass, in which the bronchial tubes may be seen abruptly terminating. This subject has been beautifully delineated in Dr. Hope's splendid work on morbid anatomy, decidedly one of the most admirable productions in this department of the age.

Inflammation of the pulmonary texture was not rare in the cases observed by the author. In twenty-three subjects the lungs were in a state of simple engorgement, or the first degree of pneumonia, and in eighteen they were more or less hepatized; the lower lobes being generally more frequently and deeply affected than the others. The mucous membrane of the bronchia sometimes retained its natural paleness, but commonly it was of a bright red color; occasionally it was thickened, and even ulcerated. Dilatation of the air tubes, with hypertrophy of all their tunics, is stated to have been very common, especially in the lower part of the lungs.

Thus far we have progressed slowly, having traveled only over thirty-eight pages of the work before us. M. Louis having omitted many important circumstances respecting the nature and origin of tubercle—from the fact that these subjects had been already ably discussed by several of his countrymen—it was deemed necessary, in order to give greater interest to this part of our review, to supply, in some degree, the deficiency. With these remarks, we proceed, secondly, to take up the consideration of the morbid anatomy of the pleuræ, larynx, the alimentary tube, the spleen, and liver; the organs most frequently implicated, according to the author, in phthisis.

Nothing was so common as *adhesions* between the lungs and their serous investments. In one hundred and twelve cases there was but a single instance in which both lungs were perfectly free in the whole of their extent. Extensive adhesions were generally associated with large excavations, and conversely, small excavations were usually connected with limited adhesions; they consisted, for the most part, of dense cellular tissue, and were probably the result of chronic inflammation. In some of the cases, the adhesions were formed by false membranes; which, in several instances, were converted into tubercular matter. An effusion of *clear fluid*, sometimes upwards of a quart in quantity, was frequently observed in the cavity of the pleuræ. This secretion came on at times very rapidly, occasionally only twenty or thirty hours before death.

One of the most valuable portions of the work is that which relates to the state of the epiglottis, larynx and trachea in phthisis. The lesions of these organs, it is true, had attracted the attention of previous observers; but until the investigations of M. Louis, our knowledge concerning them could be considered as little else than vague and conjectural. Of 102 patients, M. Louis found the epiglottis ulcerated in 18, the larynx in 23, and the trachea in 31. The ulcerations existed almost solely on the laryngeal surface of the *epiglottis*, more particularly on its inferior half, and their dimensions were from one to two lines, often larger; their edges were commonly thin, and the mucous membrane immediately adjacent more or less red. These erosions, which were remarked *twice* as often in men as in women, sometimes extended into the substance of the cartilage, destroying it partially in four cases, completely in a fifth.

The ulcerations of the *larynx* were generally deep, irregular, and from one to ten lines broad. The mucous membrane immediately around them was pale and perfectly sound, and their edges, which were of variable consistence, were sometimes lardacious, and of a greyish or whitish color. The most frequent seat of these ulcers was, first, the junction of the vocal cords, then these cords themselves, next the base of the arytœnoid cartilages, and finally the interior of the ventricles of Morgagni. In some instances one or more of the vocal cords were completely destroyed, and the base of the arytœnoid cartilages denuded, but otherwise healthy.

In the *trachea*, the ulcers were largest and most numerous in the lower half; they were of a round or oval form, a line or more in diameter, their edges flat, as if they had been scooped out with an instrument, their bottom formed by the cellular tissue, and the surrounding membrane generally of a bright red color. In most cases the ulcers exhibited a preference for the posterior portion of the tube, and here it was not unusual to find the muscular stratum exposed, and twice or three times the natural thickness. The cartilaginous rings were sometimes denuded, ulcerated, and either partially destroyed, or their texture entirely divided at one or more points. In five cases, there was complete destruction of the mucous membrane of the tube nearly through the whole extent of its fleshy portion.

"Phthisis," says M. Louis, "has been considered as one among the numerous causes of aneurism of the *heart;* but this opinion does not appear to us to be supported by facts." In the few examples in which there was hypertrophy of this organ, the thickening was entirely confined to the left ventricle, and never exceeded a few lines. In the great majority of cases the heart is stated to have been under its usual dimensions, being not more than one-half or two-thirds of the normal volume. This by no means accords with our own or the experience of other writers. In most of the consumptive patients whom we have examined—and they have not been few—the heart was found in a state of hypertrophy, sometimes both ventricles, but more commonly the left. The organ was usually of good consistence, more frequently soft than indurated; and in several instances it had contracted firm adhesions with the pericardium. The diminution of the volume of the heart is supposed by the author to have been caused rather by the great atrophy of the system than by any influence exerted by the lungs.

The *aorta* was healthy in the majority of instances, but in about one fourth of the cases it was stained red, either partially or over the whole of its extent. With one exception, this discoloration was confined to patients between the ages of twenty and thirty-two, and as it penetrated to a considerable depth into the middle tunic, M. Louis justly concludes that it must have been inflammatory. In some of the cases the caliber of the vessel was slightly contracted, as was ascertained by accurate comparative measurements.

Ulcerations of the *pharynx* and *œsophagus* were observed only in two cases; they were small, numerous, and scattered nearly uniformly over the mucous coat, which was slightly thickened in the intervening portions. These lesions were met with only in persons who died of phthisis. The internal surface of the œsophagus was frequently lined by a kind of broken up false membrane, very much like the apthous incrustations witnessed in the mouth; the cuticle beneath had disappeared, but the mucous tunic was perfectly sound. In no instance were there any symptoms indicative of these affections.

The *stomach* was frequently affected, sometimes in a variety of ways. In nine observations out of ninety-six, the organ was twice or three times its natural volume, its great curvature reaching in six of the cases down on a level with the iliac crest. This condition which is almost peculiar to phthisis, is supposed by the author to be the consequence of repeated shocks of coughing. Softening with diminished thickness of the mucous coat was present in about one fifth of the cases. It was most frequently observed in the great cul-de-sac of the stomach, often over a very large extent of surface; in the majority of instances the adjoining structure was mamillated, of a red or greyish tint, thickened, or ulcerated. Occasionally there was redness with thickening; more frequently, however, with softening. The mamillated appearance was observed in nearly one-fourth of the cases. It consisted of rounded, oval, or irregular elevations of the mucous membrane, one or two lines in diameter, of a pale greyish color, looking a good deal like the fungous granulations of an old sore. In a twelfth part of the cases there were ulcerations of the mucous tunic, generally small, few in number, and accompanied by some other lesions. In one of the patients, the ulceration was single, and two inches and a half in diameter; in another, there were as many as eighty small ones. They seldom penetrated beyond the mucous tissue, and their edges were flat and of the natural color. On the whole, M. Louis is inclined to regard phthisis as a predisposing cause of inflammation of the mucous membrane of the stomach, and that in its most intense forms, having found this organ in a sound condition only in about a fifth part of all the cases which he has analyzed.

The *duodenum* seldom deviated from the normal standard. The mucous follicles were frequently considerably augmented in volume, but not changed in structure, and in three cases out of sixty ulcerations were present. The erosions were for the most part small, not more than a line and a half in diameter, and in two of the patients they were associated with minute cysts in the liver, filled with a greenish, pulpy substance. In sixty-five persons who died of other chronic diseases, ulcerations of the duodenum occurred only in one case.

In the *small intestines* the lesions most frequently witnessed were softening, thickening, redness of the mucous tunic, small abscesses in the submucous cellular tissue, tubercular granulations, and ulcerations. Thickening was met with only in three or four cases, softening in eight; redness in thirteen. The granulations were of two kinds; sometimes they had all the characters of tubercular matter; at other times they were much harder and whiter, presenting almost the firmness and aspect of cartilage. Of the average volume of a middle sized pea, they were developed in both cases beneath the mucous coat, and almost invariably accompanied by ulceration. The *semi-cartilaginous* granulations, which were in general much more abundant than the others, were equally dispersed over the circumference of the intestine, and increased in number and size as they approached the cœcum. So long as they continued small, the mucous membrane did not seem to be affected by their presence, but when they attained the magnitude of a pea, it became usually more or less red, thickened and softened, or even destroyed at the point of union. The granulations themselves then began to decrease in size, the ravages proceeding until they were completely removed from their natural beds. The ulcers thus formed had white, opake, indurated edges, and retained almost exactly the characters of the little tumors to which they succeeded.

The *tubercular granulations* were constantly more numerous near the cœcum; in no instance did they occur in the upper part of the small bowel. Much difference prevailed in regard to their location. Sometimes they were found in the midst of an ulcerated surface, or immediately around it; upon the glands of Peyer, or in their intervals; in the interstices of the muscular fibres; or in the subperitoneal cellular tissue. These small bodies, so different in their situation from those just described, were succeeded by minute ulcers, produced by the same process as tubercular excavations of the lungs. Either separately or conjointly, these two species of granulations existed in thirty-six out of the ninety-five cases already mentioned.

Amongst the most common lesions of the small intestines were *ulcerations*. They were remarked in different proportions in seventy-eight cases, making them more than twice as frequent as the granulations, being present in nearly five-sixths of all the patients. Andral found the intestines sound only in about one-fifth of all the phthisical subjects who entered the wards of M. Lerminier during five years. (Clin. Med. vol. iii., page 175.) This proportion differs remarkably from that of Bayle, who met with this lesion in only two-thrids of his cases; but it may be readily accounted for by the greater care which Louis and Andral employed in clearing and thorougly scrutinizing the whole alimentary tract.

The size and number of these ulcerations always increased as they approached the cœcum, and in the majority of cases they were confined to the lower third or half of the tube. In their shape they were round, elliptical, circular, or linear, the frequency of their occurrence being in the order here enumerated. Their dimensions varied from a line to five or six inches; their margins, which were generally elevated and ragged, were sometimes greyish, black or pink, more rarely brownish or blackish; their bottom was generally formed by submucous cellular tissue, more or less thickened, sometimes by fleshy fibres, sometimes by peritoneum.

Besides these lesions, minute *abscesses* were occasionally found in the submucous cellular substance, about the size of a pea. They were sometimes present when there neither existed ulcerations nor tubercles in the small intestines.

"Many of the morbid alterations now described," says M. Louis, "as the softening, thickening, redness of the mucous membrane and semi-cartilaginous granulations, are common to phthisis and to a great number of other chronic and acute affections; but the semi-cartilaginous granulations are more frequent in phthisis than in any other conditions. The tuberculous granulations and ulcerations appear peculiar to this disease. We have never remarked the former except in phthisis; and if it is not rigorously correct to say that ulcerations of the small intestines, are exclusively found in this affection, exceptions are so rare that the proposition is almost literally true. Out of eighty-five cases, consisting of various chronic affections, we have only met six in which the small bowel was ulcerated." The translator, in commenting on this passage, remarks, that during the eight years which have elapsed since the publication of this work, M. Louis has not examined a *single subject* who died from a chronic disease and presented ulcerations in the small intestines, in which he did not find *tubercles in the lungs*. This, if true, must certainly be regarded as a most extraordinary fact. The circumstance, however, is almost incredible; it assuredly is not in unison with the experience of American and English pathologists. In numerous instances have we met with ulcers of the small bowel, unconnected entirely with pulmonic tubercles; at the same time we are ready to confess that we have never examined a single phthisical patient without finding ulcerations of the mucous coat of some portion or other of the alimentary tube, generally of the ileum, cœcum, or ascending and tranverse colon.

The lesions observed in the *large intestines* were similar to those of the small, with the exception of the semi-cartilaginous granulations, which were wanting. In rather more than one-fourth of the cases, there was inflammatory redness of the mucous membrane, and softening in nearly two-thirds. This lesion always occupied a large extent of surface, and in a few instances it reached through the whole of the large bowel. Tubercular granulations were present in about one-eighth of the patients; and, what is worthy of notice, they were always situated either in the centre or at the periphery of the ulcerations, never in their intervals. Ulcerations were seen seventy times out of ninety-five, making them nearly as common here as in the small intestines. Usually they were small, from three to six lines or less in diameter, rounded, elongated, or radiated, with flat, greyish, sometimes elevated and ragged margins, their base formed by the submucous cellular substance, more or less thickened, indurated and brittle. When the erosions were small they were sometimes scattered throughout the whole intestine in an almost uniform manner, but more generally their numbers diminished from the cœcum to the ascending colon, and from the transverse to the rectum, in the ratio of seventeen, eleven, eight and four. This agrees very well with our own observations. Specimens, of every grade and variety, of these kinds of lesions are preserved in the museum of morbid anatomy in the Cincinnati College.

The *lymphatic glands* were often tuberculated, the relative frequency being as follows: the mesenteric, meso-cœal, meso-colic, cervical, lumbar, and axillary. Out of 102 cases, in which they were carefully inspected, the mesenteric glands were found tuberculated in 23. The affection was seldom universal; in the generality of the cases it was most conspicuous in the glands nearest the cœcum. The duration of phthisis did not seem to exert any influence upon this tubercular deposition, as it was equally frequent in persons in whom the disease was recent, as when it was protracted for years. No symptoms were present which could be attributed to the alteration before us, either of a local or constitutional character. The cervical glands were more or less tuberculated in about one-tenth of the cases, larger than natural, and more or less vascular. M. Louis considers the tuberculization of the lymphatic glands peculiar to phthisis, and he even goes so far as to affirm that this morbid change *never* exists, after the age of fifteen, without tubercles in the lungs. This sweeping assertion has been contradicted by M. Broussais. It is certainly in opposition with the results of our best writers, and we hold it to be utterly untenable upon pathological grounds.

The *liver* was variously affected; but the most frequent, and at the same time the most remarkable alteration of this organ was the fatty transformation. M. Louis has proved its existence in one-third of his cases of phthisis, whilst he met with it only 9 times in 230 persons who died of other diseases. He has also ascertained that it is much more common among women than in men, in the ratio nearly of four to one. It was observed more frequently in the young than in the old; and in some instances it seemed to take place very rapidly, in the course of five or six weeks. The liver in this condition was of a light brownish or yellow color, speckled with red, and at times double it usual dimensions, the increase being almost invariably at the expense of the right lobe. The consistence of the organ was greatly diminished; it yielded easily to traction, and in advanced cases greased the hands, like common fat. How is this state induced? Is it owing to inflammation? The fact that it always occupies the *whole* of the liver almost precludes this idea; yet this after all is the most plausible opinion that can suggest itself. The fatty transformation is rare in this country, both in phthisis and in other maladies.

Tubercles of the liver were observed in only two cases; in a few there were numerous small cysts, from one to three lines in diameter, filled with a greenish pulpy substance; in one the middle lobe was destroyed and replaced by a large mass of hydatids. The consistence of the parenchymatous structure was variable, sometimes soft, at other times preternaturally firm, often combining induration with brittleness, but in no instance giving rise to any characteristic phenomena.

The *gall-bladder* itself was seldom morbidly affected, but its contents were frequently very dark colored and of a thick, viscid consistence, like treacle.

The *spleen* was softened, and under or above its natural volume in a great number of instances. It was tuberculized in one sixth. The tubercles were very numerous, of the size of a hemp-seed or filbert, more or less round, yellowish, opake and of a dull appearance; in a word, in all respects similar to those of the lungs; they were never encysted, and the circumjacent texture was always normal. Tubercles of this organ were not observed in persons who died of other diseases. In our own observations, this state of the spleen has been witnessed twice unconnected with phthisis.

Many examples occurred of serous effusion into the *peritoneum;* the men were equally affected as the women, and it was not more frequent where there was disease of the liver or mesenteric glands than where these structures were sound. Besides the effusion, M. Louis occasionally found a soft, yellowish, adventitious membrane, and a small quantity of thick inoderous pus, such as exists in phlegmonous abscesses. These phenomena were present in four cases, and the symptoms clearly proved that the acute peritonitis, by which they were produced, was developed only a day or two before death. In several instances the membrane was studded with tubercles.

The *urinary organs* were seldom much affected. The renal capsules contained in two instances a small quantity of concrete tubercular matter, which, as it was never observed in persons who died from other diseases, the author considers as peculiar to phthisis. The kidneys in three-fourths of the examples were perfectly sound. Occasionally they were preternaturally firm and vascular. In four cases, small cysts were developed in their interior, and in three they contained tubercular matter. The bladder, which was inspected only a few times, presented no morbid appearance.

Out of forty cases in which the *prostate gland, seminal vesicles, and excretory ducts* were scrupulously examined, the first of these organs contained tubercular matter in three, and in another it occupied the whole of them. The individual was 24 years old, by trade a tailor. The prostate gland retained its normal volume, but was almost entirely converted into hard tubercular substance. The seminal vesicles, indurated and rather larger than usual, were filled with similar matter, divided into masses by the natural intersections of the part. These intersections were hard, of a greyish color, and more than half a line thick. The excretory tubes, for about three inches, offered the resistance of dense cords, their walls being twice the ordinary thickness, and their cavity filled with firm, unsoftened, tubercular substance. This case is remarkable, and decidedly one of the most interesting on record.

M. Louis once found the inner surface of the *uterus* studded with tubercular matter, and twice he observed a small quantity of the same substance in the *ovaries*.

Though in phthisis the cerebral functions are commonly undisturbed, frequently even to the last moments of existence, M. Louis found the brain or its envelopes affected in a large number of the cases which he examined.

The cerebral arachnoid in two cases was lined by a soft, yellowish, false membrane, and in a considerable number of others it was partially thickened, opake and studded with granulations. In three-fourths of the examples there was serous effusion, either into the ventricles, on the surface, or at the base of the brain. In one-seventh, the brain was injected; in one-twentieth, softened.

No notice seems to have been taken in these examinations of the *pancreas*. How could so close and diligent an observer, as M. Louis, commit so singular an oversight? Why was this organ less worthy of consideration than some of those which he so thoroughly scrutinized? Every body seems to neglect this viscus, and yet every body admits that it must be of some use. Its morbid anatomy is less understood, certainly, than that of almost any other structure. Lombard found it tubercular in five cases of phthisis of infants.

M. Louis, at the close of this part of his work, makes the extraordinary assertion, that he has never, in a single instance, seen an organ affected with tubercles without these bodies being at the same time present in the lungs. "Their existence in these viscera seems," says he, "to be a necessary condition for their development in other parts." In reading these remarks one would scarcely suppose that the author was in earnest, and yet such would really appear to be the case. His experience upon this subject is certainly at variance, in a very eminent degree, with that of some of the most distinguished pathological anatomists of the present day, as Laennec, Andral, and Lombard. All these writers positively declare that they have repeatedly witnessed tubercles in various organs when *none* existed in the lungs; and our own observations, limited as they have been, are precisely to the same effect. In a boy, four years old, whom we examined last summer, a number of tubercles existed in the liver, but not a single one in the lungs, and in a child of twelve months, which we examined a short time since for Dr. Woodward, although the spleen was literally crowded with these bodies, none whatever could be detected in the thoracic viscera.

Part 2.—The second part opens with an account of the *symptoms* of phthisis, founded, as usual, upon the author's personal observations. In the first stage, there was generally more or less cough, with thin frothy expectoration, and pain in the side of the chest or between the shoulders. The respiratory murmur, as indicated by the stethoscope, was not sensibly changed; there was little gastric or enteric disturbance; and hœmoptysis was present in but few cases. In the second stage, the cough became more troublesome, especially during the night; the emaciation was now considerable; the sputa were of a rounded shape, with ragged edges, and of a peculiar greenish color, not unlike pea soup, sometimes streaked with opake, yellow lines, at other times with blood; the thoracic pains were often more acute than previously; hœmoptysis was pretty frequent, though in general not copious; the position of the patient in bed varied, lying sometimes on the back, sometimes on one side, sometimes on the other; the percussion in one third of the cases was dull under one clavicle, and by auscutation more or less evident pectoriloquy, with tracheal respiration, could be detected in one or more points, corresponding with the summit of the lungs. In this stage, the symptoms of gastric and laryngeal lesion were developed.

The duration of each stage was liable to considerable variation. Out of 114 cases, rather more than two-tenths died from the first to the sixth month of the disease; four tenths from the sixth to the twelfth month; rather less than a fourth from the first to the second year; and less than one-fifth from the second to the twentieth. Age seemed to have no influence in determining the duration of the disorder.

The *cough* varied much. It was generally most harassing towards the close of life. Sometimes it occurred in paroxysms, and in most cases it was worst at night. The quantity of the expectorated matter varied at different periods of the affection. In rapid cases, from ten to twenty ounces were sometimes discharged in the twenty-four hours, even in the commencement of the disease. In the second stage, it was generally less copious; sometimes, indeed, very scanty, or almost absent. After continuing for sometime, it was not unusual for the sputa to lose their greenish and opake appearance, and to become clear and vitrified, either retaining or losing their rounded form, and finally resuming their previous aspect.

*Hæmoptysis* was present in two-thirds of the cases, in 57 patients out of 87. In 25 it was copious. It sometimes preceded the cough and expectoration, and was seldom present towards the termination of the disease, when the patient was much exhausted. Sex had an evident influence on the occurrence of hœmoptysis, being more frequent in women than in men, in the proportion of three to two.

The *dyspnœa* was commonly very slight, and seldom noticed by the patient until after exercise. The oppression was referred, almost without exception, to the central part of the chest. Many of the patients were altogether exempt from pain, or only spoke of it when their attention was directed to the subject. It was ordinarily of a pleuritic character, and either seated between the shoulders or on the lateral parts of the chest, corresponding always with the adhesions of the lungs to their serous covering.

*Rigors* were present in the great majority of cases, coming on every evening, seldom at any other period. They were commonly followed by copious perspiration, and occasionally returned several times in the day, though never with any decided regularity.

One of the most frequent symptoms was diarrhœa. Out of 112 patients, only five escaped it. In some of the cases it began with the disease, and persisted until dissolution; but in most of them it did not make its appearance until the second stage, sometimes not until much later. This symptom was generally associated with and dependent upon ulceration of the alimentary canal. Rapid and universal emaciation frequently attended this condition.

*Softening* of the mucous membrane of the stomach with ulceration was indicated by nausea, vomiting, and epigastric pain, sometimes pungent and lancinating, but more generally of a dull aching character. In some instances there was a feeling as if a bar had been stretched across the region of the stomach. Inflammation and ulceration of the gastric lining were indicated by the ordinary symptoms. The mamillated state of this membrane does not seem to give rise to any characteristic phenomena. In some of the patients the appetite was merely diminished; in others, there were tenderness on pressure, nausea and vomiting.

By most physicians the state of the *tongue* is considered as indicative of that of the stomach. The fallacy of this opinion, however, has been fully shown by the recent observations of some of the French and English pathologists. In some of the phthisical cases observed by M. Louis, the organ was perfectly natural, in others, as when the disease was complicated with gastric symptoms, it was red, either entirely or at the tip or sides; and in others, again, it was covered with an albuminous exudation. The exudation, which was developed towards the close of life, occurred either in small patches, or in minute globules, which, by coalescing with each other, sometimes incrusted the whole surface of the tongue. It was easily wiped off, being not more than a fourth of a line thick, and generally re-appeared several times before death. It was invariably attended with pricking pain, heat, and redness—symptoms distinctly showing its inflammatory origin; and in the generality of instances it took place simultaneously on the tongue, lips, cheeks, gums, and occasionally even on the palate. This state of the tongue has generally been classed among the fatal phenomena of phthisis, but M. Louis has seen it almost as often in patients who recovered as in those who died.

It has been already seen that ulceration of the *epiglottis* was rather a frequent complication. This condition was indicated by a fixed pain in the upper portion of the thyroid cartilage, or between it and the hyoid bone, with difficulty of swallowing and regurgitation of fluid by the nose. The uneasiness was compared to that of a raw sore, or burn, and was present for a variable period before death. The symptoms of ulcerated larynx varied according to the seat of the disease. In general, there were more or less alteration of the voice, hoarseness sense of heat, dryness and pricking, attended sometimes with partial, at other times with complete aphony. When the ulcerations were superficial, or of small extent, there was often an entire absence of pain; but under the opposite circumstances it was distressing and continued. Ulcerations of the trachea, however numerous they might be, seldom gave rise to any symptoms—never, excepting once, to any that could be considered as pathognomonic.

The following group of symptoms is considered by M. Louis, as *diagnostic* of the first stage of phthisis: dry cough existing for a longer or shorter period, accompanied with clear, mucilaginous expectoration, pain in the side of the chest or between the shoulders, hæmoptysis at the invasion or during the progress of the cough, diminution or alteration of the respiratory murmur, and dullness of sound under one or both clavicles. When all these signs are present in the same individual, we may be sure of the existence of crude tubercles, especially if, in connexion with them, there be dyspnœa, loss of appetite, emaciation, and great sensibility to cold. In the second stage, where excavations take the place of tubercles, the diagnosis is commonly very easy, the pectoriloquy and the greenish state of the sputa furnishing never failing signs. But we have not time to dwell farther upon these points; those who are anxious to obtain more ample information upon the diagnosis of phthisis must consult the work itself, or refer to the treatise of Laennec or the manual of Collins, of Sharpe, or of Raciboski. As calculated to answer the same purpose, we would particularly recommend, while on this subject, the admirable little work on diagnosis of thoracic diseases, by Dr. W. W. Gerhard of Philadelphia; a production which should be in the library of every American physician.

Although it is a matter of much importance to be acquainted with the various *causes* of phthisis, it must be confessed that our knowledge in relation to them is any thing but satisfactory. In this case, as in many others, assertion, observes M. Louis, is much easier than proof, and the detection of error than the discovery of truth. After stating that his researches have failed in demonstrating the causes of tubercles in the lungs, the author successively examines the influence of sex, pneumonitis, pleurisy, bronchitis, dress, heriditary predisposition, and age on the production of phthisis, founding his remarks, as usual, upon personal observation. A brief analysis of the facts here developed will not be uninteresting to our readers.

Women are more prone to phthisis than men, in the proportion nearly of nine to seven. These results of the author's are fully confirmed by the researches of several recent French writers. M. Chateneuf, in a very interesting memoir, states that out of 1554 deaths from pulmonary consumption, 745 were men, 807 women. In the statistical Tables of Paris, published under the auspices of M. Charbol, in 1830, we find that out of 9542 cases of this disease, 5582 were women,

3960 men. Similar testimony has been furnished by other writers, both French and English; so that there would seem to be little doubt as to the greater liability of the female sex.

The development of phthisis seems to be but little influenced by the presence of pneumonitis, bronchitis or pleurisy. "The opinion then," says M. Louis, "that pulmonary tubercles are the result of chronic inflammation of the bronchial mucous membrane, pulmonary parenchyma or pleura, on whatever theory it may be supported, is quite unsatisfactory; the preceding results cannot be set aside, except by a large series of observations, which shall prove that the proportion we have established originated from a purely accidental combination of facts." In another paragraph, he observes: "not only is the influence of these diseases in the development of phthisis not demonstrated, but our observations induce us to suppose its existence imaginary, or at least restricted within very narrow limits."

That *dress* exercises an unfavorable, and oftentimes highly disastrous influence upon the human frame, the experience not only of the profession but of mankind at large sufficiently attests. Tight lacing, on the part of the young female especially, must be extremely injurious, from the pressure which it exerts upon the tender and delicate organs of the chest, always stinting their growth, and frequently leading to structural derangement. Even when the body has attained its full expansion, stays can seldom be worn without more or less detriment. But we have not time to dwell upon this subject; those who may desire further information respecting it, will do well to consult the excellent paper on tight lacing, by the lamented Godman, a name dear to every American physician. M. Louis seems inclined to ascribe but little influence to this source upon the production of phthisis, from the fact probably that most of those who died from this disease under his notice were females who had been brought up in the country, and had only been accustomed to the use of stays after the body had attained its full growth.

M. Louis has not collected any facts in favor of the *hereditary* nature of phthisis, though one-tenth of the patients were descended from consumptive parents. He does not, however, deny this sort of influence; but is of opinion that, before the question can be finally solved, extensive statistical tables of mortality will be necessary, comparing an equal number of patients born of phthisical parents with those of an opposite condition. The translator, Dr. Cowan, in an interesting note appended to this section of the work, makes the following apposite remark. "In general terms, it may be stated, that children are hereditarily predisposed to phthisis in proportion as their general health is enfeebled, whatever may be the cause, and that attention to this fact is of more practical importance to the physician, than the knowledge, whether tubercular disease did or did not exist on the part of the parents."

The period of life most liable to phthisis, is between twenty and forty. This fact, which seems to have been clearly indicated by Hippocrates and his immediate descendants in medicine, has been amply confirmed by the united experience of hundreds of modern physicians. Out of the 123 patients observed by M. Louis, not less than 72 died during this interval. These results are confirmed by those of Dr. Morton, Bayle, Darwall, and other writers. In 281 phthisical persons who died in the Philadelphia Alms-house, 193 perished under forty years of age. Forty-two died between the ages of forty and fifty; twenty between fifty and sixty; twelve between sixty and seventy; three between seventy and eighty; two between eighty and ninety; one between ninety and a hundred.

Dr. Lombard, of Geneva, has found that children are most prone to tubercles between the ages of four and five. Infants are sometimes born with this disease. Of this, Chaussier and Billard have each witnessed cases. Professor Rives, of the Cincinnati College, a few years ago, met with a case of tubercles in a child six weeks old, some in a concrete, others in a softened state; and in a child less than three months of age, which we examined last winter for Dr. Woodward of this city, similar phenomena were observed.

It would seem rather singular at first sight, that a work embracing so large an amount of pathological matter, should contain so meagre an account of the *treatment* of this prevalent disease. This, however, so far from forming an objection to the work, constitutes in our opinion one of its most valuable features. M. Louis, as has been already seen, is a matter-of-fact man; he has carefully studied nature, and, like a true philosopher, varies his treatment according to the indications, founded on the state of the functions, and the different complications occurring in the progress of the principal disease.

The decoction of Iceland moss, and the gum potion with the occasional employment of small doses of syrup of poppies, to allay the cough and procure sleep, constituted the principal means in the early stage of the disease, when attended by thoracic complication. When fever was present without local inflammatory symptoms, as was frequently the case in the second stage, demulcent drinks, and an infusion of pectoral flowers, with rice and milk, were ordered; and under this treatment the violence of the fever is said to have gradually abated, followed by a general improvement in all the functions. If the symptoms happened to be of an acute character, general and local bleeding were sometimes resorted to, though usually with little or no benefit. Syrup of poppies, opium, acetate of morphia, and extract of belladonna were prescribed to allay the cough; the opium occasionally produced pungent pain in the throat, with dryness of the fauces, and on this account was obliged sometimes to be omitted. The pneumonic and pleuretic complications were best relieved by venesection, leeches, and blisters.

Venesection was also employed in cases of hœmoptysis, but commonly without much relief. The same results followed the application of blisters. When the hœmorrhage was slight, reliance was placed mainly upon demulcent drinks, the hand and foot bath, enemeta, and light diet, joined occasionally with a very small bleeding from the arm.

The dyspnœa was sometimes releived by blisters, but in general they failed in effecting this end. Local bleeding and vesication were tried in some of the cases of ulcerated larynx, without success. The author seems to think well at certain periods of the disease of the application of medicated vapors, but judging from his silence on the subject, it is more than probable that he did not employ them.

Where the rigors were very troublesome and regular in their recurrence, the sulphate of quinine was administered, under the influence of which they either greatly diminished or totally disappeared. The acetate of lead was given in six cases to arrest the profuse night sweats, but although the dose was gradually increased to twelve or fifteen grains daily, in only one instance did it succeed.

The gastric symptoms, which so often existed, were endeavored to be overcome by leeches, demulcent drinks, seltzer water, and opium. To these, if the thirst was urgent, was added a solution of gum or tartaric syrup. All drinks, however, soon inspired disgust, producing a sense of weight and oppression in the epigastrium.

The diarrhœa, as has been already seen, was often troublesome and debilitating. To relieve this unpleasant symptom M. Louis resorted to the use of diascordium, catechu, and opium, both in the early and the more advanced stages of the complaint. The diet consisted of the most bland and unirritant substances, with rice and gum water for drink.—Ratanhia root was also tried, but like the other remedies just mentioned, it was rarely attended with any decidedly good effects.

Such is a faithful outline of the treatment of phthisis employed by M. Louis. The plan, it is obvious, is rather palliative than curative—it recommends no Herculean means—it boasts of no specific—it does not kill the sufferer by copious depletion—it does not send him on the ocean or in foreign countries to die away from his friends, as is so often practiced by the American physician—but it aims at sustaining tottering nature, and lets the patient perish only when he can no longer swallow rice water and pectoral ptisans. This after all is the best way, the true philosophy of treating a disease which has uniformely baffled the best efforts of the sages of the profession from the earliest ages of the world until the present moment. Incurable as this disease now is, who knows but that it may one day come perfectly under the control of the physician? Shall we relinquish our efforts because our skill has hitherto been of no a vail? No! let us study nature; and following the example of the illustrious Louis, contemplate her in her various aspects of health and disease; let us investigate animal chemistry, general and morbid anatomy, and physiology, and who knows but the time may come when our acquaintance with the nature of tubercles may be such as to enable us to devise some plan which shall prevent their formation, or which, when formed, shall successfully oppose their softening?

Physicians have kept up a crusade from time immemorial against disease; they have established colleges and universities for teaching discriptive anatomy, chemistry, materia medica, and the practice of medicine; they have written books, without number, to point out the best method for distinguishing the various disorders of our frame; but strange to say few seem to have ever reflected on the propriety of indoctrinating the student into the first principles of his profession, of making him acquainted at the very outset of his inquries with the foundation of all medical science—the rudiments of morbid anatomy—the alterations of structure induced by disease. No where is this more eminently true, more culpably overlooked, than in our own country. While pathological anatomy is taught—and taught too with all possible zeal and earnestness—in every respectable institution in Great Britain and on the continent of Europe, it is almost wholly neglected in our own, the very word indeed being seldom heard, or if heard, only as it were by accident, within their walls. Will any one pretend to say that this is as it should be? Is this the method of instruction pursued in other departments of human knowledge? Is it philosophical to study the more complex parts of a science before the student has formed an acquaintance with its first principles? No one will affirm that it is, and yet this folly is daily committed by the young disciple of medicine, a branch of knowledge in which above all others a course of this kind must be eminently injurious. The fact is, we teach our students to run before they have learned to walk; to read, before they have learned to spell; we allow them to practice their profession before they have acquired a thorough insight into its fundamental principles; we should rather have said, before they know any thing at all about the matter.

What are the rudiments of a sound medical education? Every one will answer anatomy, but surely something more than mere discriptive anatomy; something more than a knowledge of the origin and insertion of the muscles, the relative situation of the arteries, the arrangement of the fasciæ, and the bumps of the bones. Let every candid man reflect, and tell us if this branch of the subject is not, in comparison with some others, of but partial utility; if it be not calculated rather to benefit the surgeon—the mere operator—the hewer off of limbs against time—than the real physician? What does it avail the practitioner of medicine and obstetrics if he have ever so good a knowledge of descriptive anatomy, and yet be ignorant of the very tissues which constitute the basis of the human fabric; of the various textures as they appear in health and under the tortures of inflammatory action, chemical irritants, or mechanical injury. We assert it boldly and without the fear of successful contradiction, that general and morbid anatomy are the foundation—the very corner stone—of a sound medical education; and we add, moreover, that without a competent knowledge of them, no man, whatever may be his qualifications in other respects, should be permitted to practice medicine; any more than the sailor should be allowed to carry passengers in his vessel without having a rudder and compass to guide him across the fathomless deep. But where shall the student look for information upon these two kindred subjects? Must he go to Paris, to London, Dublin, or Berlin, or can he obtain all he may wish in Philadelphia, she who proudly arrogates to herself all the science and perfection of a hundred medical schools? Shall he visit the halls of a Shippen, a Rush, and a Physick? They will re-echo not the knowledge he has come to seek. Shall he go to her young sister, she who nobly strives with her for the ascendency, and there receive the lessons which fain he would learn? Her halls, also, are silent—the voice of general and morbid anatomy is not heard—her sons, too, receive not the rudiments of a sound medical education. The names of Bichat, and of Andral, of Louis, Meckel, Carswell, and the whole host of distinguished pathological anatomists of Europe, remain unknown to them, and their works are literally sealed books. Why do these two schools, first in point in regard to the number of their students, though we sincerely believe not so in point of talents—in the country—abolish the bastard chair of the institutes of medicine, and substitute something more solid and substantial in its stead? Who hears of this professorship in France, the land of general and morbid anatomy, the fountain-head of those mighty streams of science, which are so rapidly, so successfully undermining the sandy shores of nosological medicine? We repeat it, it is high time that this chair should be abolished; it is unfit to be retained in any medical school; it is a play thing of the fancy; the cloak of ignorance of a former age. The nineteenth century deals in realities; it delights in showing things as they are, not as they are conjectured to be. General and morbid anatomy are certain sciences, admitting of clear and satisfactory demonstration, and as such they should hold an elevated rank in every school of medicine, sensible of the obligations which are due to society—to suffering humanity. Medicine, as it is now taught in the United States, is indeed "the field of the slothful that is all overgrown with thorns and nettles." To weed it requires time and talents; our hands must not refuse to labor, neither our eyes to see. "*Observation must be our motto.*"

But we must bring this review, already extended far beyond what we at first designed, to a close. In doing so, we can conscientiously declare, that we have never read a work which has given us more pleasure in the perusal, or added more to our instruction. We have endeavored to present to the reader an outline of its most prominent and interesting features, and to such as cannot purchase the book we hope our analysis will be equally acceptable and edifying. In our remarks we have strictly limited ourselves to the researches of M. Louis, having barely alluded in one or two places to the notes of Dr. Cowan, the elegant translator. Many of these notes are of a high order, and deserve an attentive perusal. Dr. Bowditch, the American editor, has also appended some valuable information, but he merits the thanks of the profession chiefly for the handsome manner in which the work has been got up by his Boston publishers. Altogether, it is one of the neatest specimens of typography that we have ever witnessed in any medical treatise, either American or European.

